# Small Extracellular Vesicles Containing miR-34c Derived from Bone Marrow Mesenchymal Stem Cells Regulates Epithelial Sodium Channel via Targeting MARCKS

**DOI:** 10.3390/ijms23095196

**Published:** 2022-05-06

**Authors:** Yu Hua, Aixin Han, Tong Yu, Yapeng Hou, Yan Ding, Hongguang Nie

**Affiliations:** Department of Stem Cells and Regenerative Medicine, College of Basic Medical Science, China Medical University, Shenyang 110122, China; huaxiaoyu0320@163.com (Y.H.); cpuaixin@163.com (A.H.); 0909pippoyu@sina.com (T.Y.); 13314221035@163.com (Y.H.); dingyan548@163.com (Y.D.)

**Keywords:** mesenchymal stem cells, small extracellular vesicles, acute lung injury, epithelial sodium channel, miR-34c

## Abstract

Epithelial sodium channel (ENaC) is a pivotal regulator of alveolar fluid clearance in the airway epithelium and plays a key role in the treatment of acute lung injury (ALI), which is mainly composed of the three homologous subunits (α, β and γ). The mechanisms of microRNAs in small extracellular vesicles (sEVs) derived from mesenchymal stem cell (MSC-sEVs) on the regulation of lung ion transport are seldom reported. In this study, we aimed at investigating whether miR-34c had an effect on ENaC dysfunction induced by lipopolysaccharide and explored the underlying mechanism in this process. Primarily, the effect of miR-34c on lung edema and histopathology changes in an ALI mouse model was investigated. Then the uptake of PKH26-labeled sEVs was observed in recipient cells, and we observed that the overexpression of miR-34c in MSC-sEVs could upregulate the LPS-inhibited γ-ENaC expression. The dual luciferase reporter gene assay demonstrated that myristoylated alanine-rich C kinase substrate (MARCKS) was one of target genes of miR-34c, the protein expression of which was negatively correlated with miR-34c. Subsequently, either upregulating miR-34c or knocking down MARCKS could increase the protein expression of phospho-phosphatidylinositol 3-kinase (p-PI3K) and phospho-protein kinase B (p-AKT), implying a downstream regulation pathway was involved. All of the above suggest that miR-34c in MSC-sEVs can attenuate edematous lung injury via enhancing γ-ENaC expression, at least partially, through targeting MARCKS and activating the PI3K/AKT signaling pathway subsequently.

## 1. Introduction

Acute lung injury (ALI) is caused by the direct or indirect damage of alveolar epithelium and capillary endothelial cells, resulting in diffuse alveolar interstitial edema [1,2,3]. Na^+^ is the primary driving force in the generation of osmotic gradient [4], which is transported by the epithelial sodium channel (ENaC) on the apical side and then exported by the Na^+^/K^+^-ATPase pump on the basolateral surface of alveolar type 2 epithelial (AT2) cells, thereby facilitating the removal of edema fluid from alveolar spaces [5,6]. The upregulation of ENaC expression has a protective effect on ALI by relieving lung edema [7].

Mesenchymal stem cells (MSCs) were originally identified in the bone marrow by Friedenstein et al. in 1968; it could thereafter be isolated from various tissues (umbilical-cord blood, Wharton’s jelly, placenta, adipose tissue and so on) [8,9,10]. They represent a class of adult stem cells which possess the following abilities in multipotential differentiation, self-renewal, immunoregulation and anti-inflammation [11,12,13]. Moreover, the therapeutic effects of MSCs in ALI model induced by lipopolysaccharide (LPS) have been reported to be related with the release of paracrine soluble factors in lung microenvironment, including small extracellular vesicles (sEVs) [14,15], which are secreted by multiple cells with a diameter of about 30–100 nm, carrying microRNAs (miRNAs), RNA, protein and other bioactive substances [16]. As one of the pivotal secretory substances of MSCs, sEVs have the similar effects with parental MSCs [17]. However, compared with MSCs, sEVs derived from MSC (MSC-sEVs) are more stable and may provide an alternative approach for the treatment of ALI [18].

In recent years, great efforts have been made aiming at exploration of sEV contents, as well as the underlying molecular mechanisms about intercellular communication. MiRNAs, small non-coding RNA molecules with approximately 20–24 nucleotides in length, can regulate the gene expression post-transcriptionally and physiological functions of target cells via binding to the 3′ noncoding regions of mRNAs when transferred by sEVs [19,20]. Therefore, miRNAs offer new insights in the field of gene regulation and have become a therapeutic option in many diseases [21]. Evidence is increasing that sEVs containing miRNAs secreted by donor cells can be taken up by recipient cells to regulate the expression of downstream target genes, which can influence diverse biological processes in cells [21,22].

In our previous research, we identified that sEVs of bone-marrow-derived MSC (BMSC-sEVs) could increase the expression of ENaC and relieve lung edema in ALI mice. We speculated that BMSC-sEVs may exert protective effect on ALI through transferring miRNAs. Previous studies demonstrated that miR-34c was found in BMSC-sEVs [23], and it was shown to be protective in the treatment of ALI, but its potential molecular mechanism is unclear and still needs future study. Therefore, we speculate that BMSC-sEVs may regulate ENaC through miR-34c, thereby alleviating ALI [24]. Moreover, researchers have demonstrated that MARCKS is the potential target of miR-34c and possibly regulates the phosphatidylinositol 3-kinase/protein kinase B (PI3K/AKT) signal pathway [24,25,26]. As one of three main subunits of ENaC, γ-ENaC is mainly responsible for binding anionic phospholipid phosphates, including phosphatidylinositol 4,5-bisphosphate (PIP2) [27,28], which can be sequestered and presented at the lipid membrane by MARCKS, implying the possible involvement of MARCKS and PI3K/AKT in regulating γ-ENaC [29]. Here we aim to verify whether miR-34c in BMSC-sEVs act to attenuate lung edema via upregulating γ-ENaC expression through targeting MARCKS and activating the PI3K/AKT signal pathway subsequently; the evidence of this would provide more information that could lead to the discovery of a novel therapeutic strategy for ALI.

## 2. Results

### 2.1. MiR-34c Attenuated Lung Edema and Histopathology Changes in ALI Mouse Model

**Figure 1:**
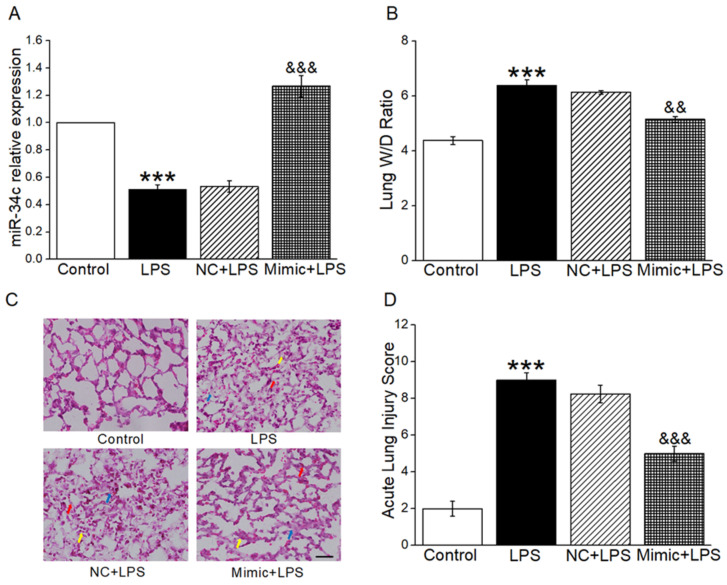
MiR-34c attenuated lung edema and histopathology changes in ALI mouse model. (**A**) The miR-34c level in mouse lung treated with miR-34c mimic by qRT-PCR assay. MiR-34c level in control (Control), LPS (LPS, 24 h, 5 mg/kg), miR-34c mimics negative control (NC, 72 h, 2 mg/kg) + LPS (24 h, 5 mg/kg) and miR-34c mimics (Mimic, 72 h, 2 mg/kg) + LPS (24 h, 5 mg/kg). The Relative level of miR-34c was calculated as miR-34c/U6 ratios (*n* = 3–4). (**B**) The W/D ratio change in LPS-treated mice (*n* = 4). (**C**) The effect of miR-34c on histopathology changes was assessed by HE staining (×400). Scale bar = 50 μm. (**D**) Acute lung injury scores were recorded from 0 (no damage) to 16 (maximum damage). Note: *** *p* < 0.001, compared with Control group; ^&^^&^ *p* < 0.01, ^&&&^ *p* < 0.001, compared with NC + LPS group.

To clarify the function of miR-34c in ALI mouse model, miR-34c mimic was injected into mice through the tail vein. As shown in the Figure 1A, the mice receiving the miR-34c exhibited higher miR-34c levels by qRT-PCR assay. In order to investigate the effect of miR-34c on the removal of edematous fluid in vivo, the W/D ratio was measured in an ALI mouse model. As expected, miR-34c significantly attenuated the increased W/D ratio stimulated by LPS (Figure 1B), which demonstrated that miR-34c could relieve lung edema in ALI mice. Finally, HE staining was performed to study the effects of miR-34c on histopathology changes in the lung tissue. As presented in Figure 1C, the lung tissue in LPS group was significantly damaged, with increased thickness of the alveolar wall (red arrow), inflammatory cells infiltration (yellow arrow) and hyaline membrane (blue arrow). The histopathology changes were alleviated in an LPS-induced ALI mouse model after miR-34c injection, as evidenced by the values of the ALI score shown in Figure 1D.

### 2.2. MiR-34c Upregulated LPS-Inhibited γ-ENaC Protein Expression in H441 and AT2 Cells

**Figure 2:**
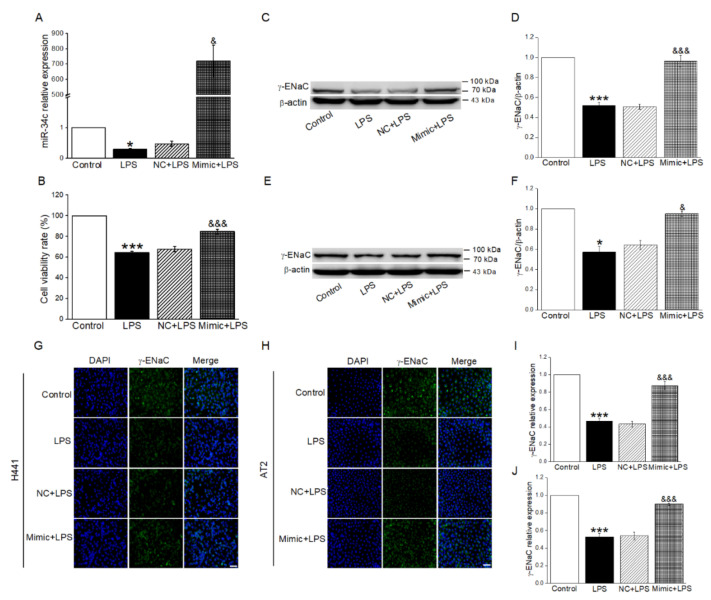
MiR-34c upregulated LPS-inhibited γ-ENaC protein expression in H441 and AT2 cells. (**A**) MiR-34c relative expression level by qRT-PCR assay in H441 cells treated with LPS for 24 h after the transfection with negative control (NC) or miR-34c mimic (Mimic) for 48 h. The normal medium (Control), LPS (10 μg/mL, 24 h, LPS), NC plus LPS (48 h, NC + LPS) and Mimic plus LPS (48 h, Mimic + LPS) group were performed in H441 cells. The relative level of miR-34c was calculated as miR-34c/U6 ratios (*n* = 4). (**B**) MiR-34c enhanced the cell viability in LPS-treated H441 cells by CCK-8 cell viability assay (*n* = 4). (**C**,**E**) Representative blots of γ-ENaC protein in Control, LPS, NC + LPS, and Mimic + LPS group in H441 and mouse AT2 cells, respectively. (**D**,**F**) Graphical representation of data obtained from Western blot and quantified through gray analysis (γ-ENaC/β-actin) (*n* = 4). (**G**,**H**) Immunofluorescent detection of γ-ENaC protein in H441 cells and AT2 cells. Nuclei were counterstained by 4,6-diamidino-2-phenylindole (DAPI, blue; left panel) and γ-ENaC (green; middle panel). The merged images are seen (right panel). Scale bar = 50 μm. (**I**,**J**) Graphical representation of data obtained from immunofluorescent detection and quantified through gray analysis in H441 cells and AT2 cells (*n* = 4). Note: * *p* < 0.05, *** *p* < 0.001, compared with Control group; ^&^ *p* < 0.05, ^&&&^ *p* < 0.001, compared with NC + LPS group.

In order to verify whether miR-34c could exert effect on the ENaC expression after LPS treatment, we transfected miR-34c mimic (Mimic) into H441 cells. As shown in Figure 2A, we found that LPS significantly reduced the expression of miR-34c in H441 cells (vs. Control group). Conversely, the level of miR-34c was increased in LPS-treated cells after the transfection of miR-34c mimic. Moreover, we found that cell viability was reduced by the LPS administration when compared with the Control group; however, this reduction could be reversed by miR-34c (Figure 2B). Subsequently, the Western blot results showed that the expression of γ-ENaC was reduced by LPS administration in H441 cells (vs. Control group), while the transfection of miR-34c mimic displayed an opposite result (vs. NC + LPS group), as shown in Figure 2C,D. Similar results were shown in AT2 cells (Figure 2E,F). Based on the above results of the Western blot, we further identified whether miR-34c exerted the same effect on the FITC-labeled γ-ENaC expression by immunofluorescence detection in H441 and AT2 cells. Moreover, γ-ENaC located in cell membrane exhibited green fluorescence in H441 and AT2 cells, whereas the nucleus was performed in blue fluorescence (Figure 2G–J). As expected, the fluorescence intensity of γ-ENaC greatly decreased in H441 and AT2 cells after LPS treatment as compared with the Control group, supporting the observation that the protein expression of γ-ENaC was reduced. On the contrary, the γ-ENaC protein expression in LPS-treated cells was significantly enhanced after miR-34c overexpression when compared with the transfection of blank plasmid. The above data demonstrated that miR-34c overexpression could alleviate the decrease of ENaC expression caused by LPS.

### 2.3. sEVs Derived from BMSCs Were Taken Up by Recipient Cells

**Figure 3:**
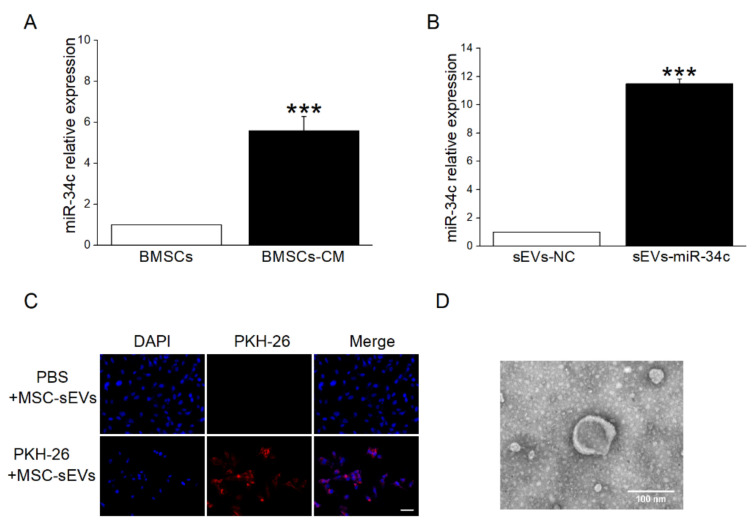
The sEVs derived from BMSCs could be taken into recipient cells. (**A**) The qRT-PCR analysis of miR-34c levels in BMSCs and BMSCs-CM (*n* = 4). (**B**) MiR-34c levels in miR-34c-overexpressing sEVs (sEVs-miR-34c) or empty vector sEVs (sEVs-NC) (*n* = 4). (**C**) Immunofluorescent detection of sEVs labeled with PKH26 after incubation with H441 cells for 20 h. Nuclei were counterstained by DAPI (blue; left panel), and the representative staining images are shown in PKH26-labeled sEVs (red; middle panel). The merged images are seen (right panel). Scale bar = 50 μm. (**D**) Morphology of BMSC-sEVs observed under TEM. Scale bar = 100 nm. Note: *** *p* < 0.001, compared with BMSCs group and sEVs-NC group.

To further test our hypothesis that miR-34c from BMSC-sEVs could upregulate γ-ENaC expression through miR-34c, we initially detected the miR-34c level in BMSCs and BMSCs-CM, respectively. The results showed that miR-34c in BMSCs-CM was expressed at a higher level than in BMSCs (Figure 3A). Additionally, compared with the sEVs derived from empty vector BMSCs (sEVs-NC), miR-34c was overexpressed significantly in the sEVs derived from miR-34c-transfected BMSCs (sEVs-miR-34c) analyzed by qRT-PCR assay (Figure 3B). More important, the sEVs labeled with PKH26 (red fluorescence) were found in co-cultured H441 cells (Figure 3C), suggesting that BMSC-sEVs could be taken up by recipient cells. In addition, TEM showed sEVs with closed round vesicles and a typical central depression (Figure 3D).

### 2.4. Overexpressing miR-34c in BMSC-sEVs Attenuated LPS-Inhibited γ-ENaC Expression in H441 and AT2 Cells

**Figure 4:**
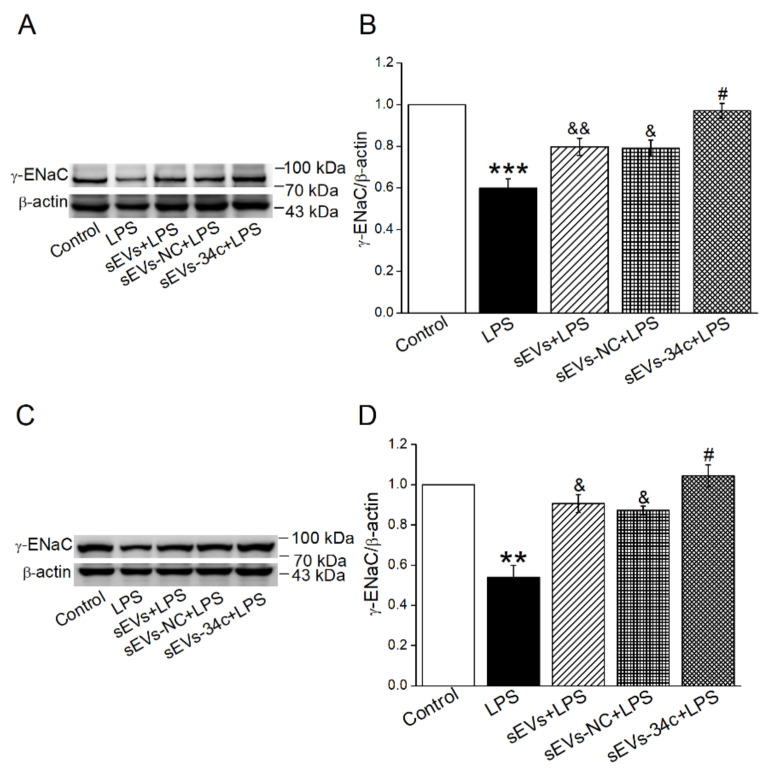
Overexpressing miR-34c in BMSC-sEVs attenuated LPS-inhibited γ-ENaC expression in H441 and mouse AT2 cells. (**A**,**C**) Representative blots of γ-ENaC protein in Control, LPS (LPS, 24 h, 10 μg/mL), sEVs (20 h, 20 μg/mL) +LPS (24 h, 10 μg/mL), sEVs -NC (20 h, 20 μg/mL) + LPS (24 h, 10 μg/mL) and sEVs -34c (20 h, 20 μg/mL) + LPS (24 h, 10 μg/mL) group in H441 and AT2 cells, respectively. (**B**,**D**) Graphical representation of data obtained from Western blot and quantified through gray analysis (γ-ENaC/β-actin) (*n* = 5). Note: ** *p* < 0.01, *** *p* < 0.001, compared with Control group; ^&^ *p* < 0.05, ^&&^ *p* < 0.01, compared with LPS group; ^#^ *p* < 0.05 compared with sEVs -NC + LPS group.

To investigate the influence of miR-34c secreted by BMSC-sEVs on the protection of ALI, we used the Western blot method to explore the γ-ENaC expression in LPS-treated H441 and AT2 cells. The protein expression of γ-ENaC in the LPS-treated group was significantly reduced (vs. Control group), but then it was upregulated after BMSC-sEVs treatment (sEVs + LPS). We also proved that the γ-ENaC was highly expressed in the LPS-treated H441 cells which were co-cultured with the miR-34c-overexpressing sEVs (sEVs-34c + LPS), compared with the empty vector sEVs (sEVs-NC + LPS). Additionally, there was no significant difference between the groups of sEVs + LPS and sEVs-NC + LPS (Figure 4A,B). Similar results were shown in AT2 cells (Figure 4C,D). These data confirmed that miR-34c in BMSC-sEVs could upregulate γ-ENaC expression, which was contrary to LPS administration.

### 2.5. MiR-34c Increased γ-ENaC Expression by Directly Targeting MARCKS

**Figure 5:**
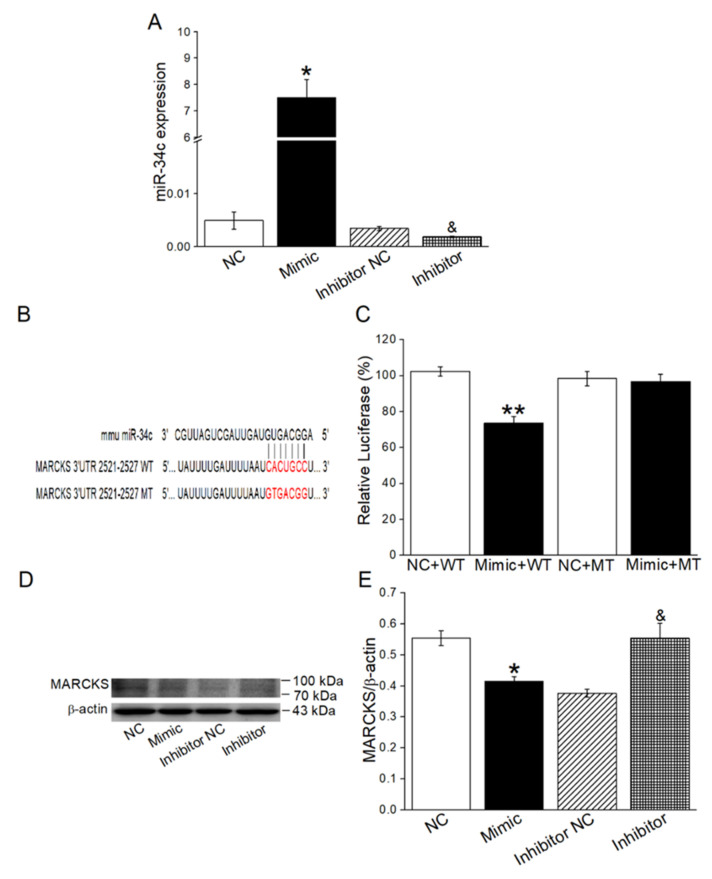
MiR-34c could bind directly to MARCKS. (**A**) Transfection efficiency of miR-34c in H441 cells (*n* = 4). (**B**) The potential binding sites for miR-34c on the 3′-UTR of MARCKS. (**C**) Dual luciferase assay for miR-34c binding with MARCKS. H441 cells were co-transfected with miR-34c mimic negative control (NC) or miR-34c mimic (Mimic) together with pmirGLO-MARCKS (WT or MT) for 24 h (*n* = 4). (**D**) Representative Western blot measurement of MARCKS protein expression in H441 cells transfected with miR-34c mimic/inhibitor. (**E**) Graphical representation of data obtained from Western blot assays for MARCKS and quantified through gray analysis (MARCKS/β-actin) (*n* = 4). Note: * *p* < 0.05, compared with NC group; ** *p* < 0.01, compared with NC + WT group; ^&^ *p* < 0.05, compared with Inhibitor NC group.

Previous studies had reported that activation of MARCKS-like protein-1 could reduce ENaC activity [30]. Moreover, potential miR-34c targets were predicted by using in silico approaches, and according to the bioinformatic website prediction, MARCKS might be a potential target of miR-34c. To confirm whether MARCKS is a direct target of miR-34c and has the intermediate effect between miR-34c and γ-ENaC, the dual luciferase target gene assay and Western blot were used in H441 cells. The transfection efficiency of miR-34c was first verified by qRT-PCR assay in H441 cells (Figure 5A), which were transfected with miR-34c mimic (Mimic) or miR-34c mimic negative control (NC), respectively, and we subsequently found that the relative luciferase activity of PGLO-MARCKS-wild type (WT) + Mimic group was significantly downregulated compared with the WT + NC group. In addition, the relative luciferase activity between PGLO-MARCKS-mutant (MT) + Mimic group and MT + NC group showed no significant difference (Figure 5B,C). Additionally, the expression of MARCKS in the Mimic group was significantly reduced when compared with the NC group. On the contrary, the miR-34c inhibitor increased MARCKS expression compared with miR-34c inhibitor negative control (Inhibitor NC) group (Figure 5D,E). All of the above results showed that miR-34c could directly bind to MARCKS and effectively inhibit MARCKS expression in H441 cells.

### 2.6. MiR-34c Activated PI3K/AKT Signaling Pathway by Inhibiting MARCKS

**Figure 6:**
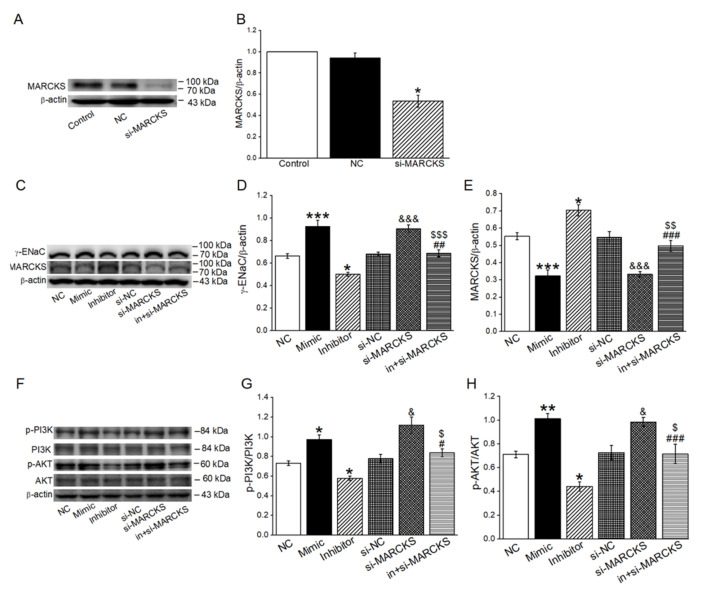
MiR-34c and MARCKS regulated γ-ENaC through PI3K/AKT pathway. (**A**) Representative Western blot bands of MARCKS protein expression in H441 cells transfected with si-MARCKS. (**B**) Graphical representation of data obtained from Western blot assays for MARCKS and quantified through gray analysis (MARCKS/β-actin) (*n* = 4). (**C**) Representative Western blot bands of γ-ENaC and MARCKS in H441 cells transfected with miR-34c mimic, miR-34c inhibitor and si-MARCKS, respectively. (**D**,**E**) Graphical representation of data obtained from Western blot assays for γ-ENaC protein and MARCKS protein. Bands were quantified by using gray analysis (γ-ENaC/β-actin and MARCKS/β-actin) (*n* = 5). (**F**) Representative Western blot bands of p-PI3K/PI3K and p-AKT/AKT in H441 cells transfected with miR-34c mimic, miR-34c inhibitor and si-MARCKS, respectively. (**G**,**H**) Graphical representation of data obtained from Western blot assays for p-PI3K/PI3K and p-AKT/AKT. Bands were quantified by using gray analysis (p-PI3K/PI3K and p-AKT/AKT) (*n* = 5). Note: * *p* < 0.05, ** *p* < 0.01, *** *p* < 0.001, compared with negative control (NC); ^&^ *p* < 0.05, ^&&&^ *p* < 0.001, compared with si-NC; ^#^ *p* < 0.05, ^##^ *p* < 0.01, ^###^ *p* < 0.001, compared with miR-34c inhibitor (Inhibitor); ^$^ *p* < 0.05, ^$$^ *p* < 0.01, ^$$$^ *p* < 0.001, compared with si-MARCKS.

In order to verify whether PI3K/AKT was a downstream pathway of MARCKS in the miR-34c regulating ENaC, we measured the γ-ENaC, MARCKS, p-PI3K/PI3K and p-AKT/AKT protein expression in H441 cells transfected with miR-34c mimic (Mimic), miR-34c inhibitor (Inhibitor) and MARCKS-siRNA (si-MARCKS), respectively. The siRNA transfection efficiency was identified by the Western blot method, and we found that the MARCKS protein expression was efficiently reduced (Figure 6A,B). The expression of γ-ENaC, p-PI3K and p-AKT was obviously upregulated after the transfection of the miR-34c mimic or si-MARCKS, while an opposite result was found in the Inhibitor group and reversed by co-transfection with si-MARCKS (in + si-MARCKS). There was no significant difference among the levels of PI3K and AKT in each group. Moreover, MARCKS expression showed an opposite direction in each group compared with γ-ENaC expression (Figure 6C–H). Hence, the upregulation of miR-34c or downregulation of MARCKS could activate the PI3K/AKT signaling pathway, which might be involved in the miR-34c regulation of γ-ENaC.

### 2.7. MiR-34c Activated the Inhibition of PI3K/AKT Signaling Pathway in LPS-Treated Cells

**Figure 7:**
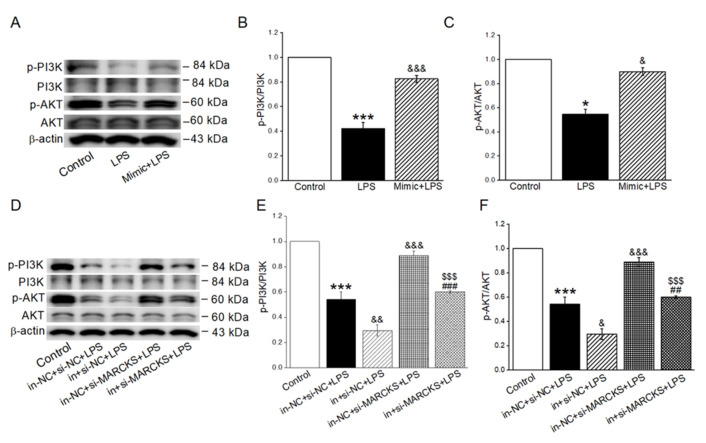
MiR-34c activated the inhibition of PI3K/AKT signaling pathway in LPS-treated cells. (**A**) Representative Western blot bands of p-PI3K/PI3K and p-AKT/AKT in LPS-treated H441 cells transfected with miR-34c mimic. (**B**,**C**) Graphical representation of data obtained from Western blot assays for p-PI3K/PI3K and p-AKT/AKT. Bands were quantified by using gray analysis (p-PI3K/PI3K and p-AKT/AKT) (*n* = 4). (**D**) Representative Western blot bands of p-PI3K/PI3K and p-AKT/AKT in LPS-treated H441 cells transfected with miR-34c inhibitor and si-MARCKS. (**E**,**F**) Graphical representation of data obtained from Western blot assays for p-PI3K/PI3K and p-AKT/AKT. Bands were quantified by using gray analysis (p-PI3K/PI3K and p-AKT/AKT) (*n* = 4). Note: * *p* < 0.05, *** *p* < 0.001, compared with Control; ^&^ *p* < 0.05, ^&&^ *p* < 0.01, ^&&&^ *p* < 0.001, compared with LPS and in-NC + si-NC + LPS; ^##^ *p* < 0.01, ^###^ *p* < 0.001, compared with in + si-NC + LPS; ^$$$^ *p* < 0.001, compared with in-NC + si-MARCKS + LPS.

To explore the possible mechanisms that miR-34c upregulated γ-ENaC expression in LPS-treated H441 cells, we further investigated the pivotal proteins of PI3K/AKT signaling pathway. The p-PI3K and p-AKT in the LPS group were significantly suppressed (vs. Control group), but this could be reversed by the administration of miR-34c mimic (Figure 7A–C). Moreover, we also assessed the effect of miR-34c inhibition or MARCKS knockdown on the p-PI3K and p-AKT expression in LPS-treated cells. As shown in Figure 7D–F, miR-34c inhibition significantly decreased the protein levels of p-PI3K and p-AKT expression compared with the LPS group, whereas MARCKS knockdown showed an opposite result. Additionally, the changes of p-PI3K and p-AKT expression in LPS-treated cells transfected with miR-34c inhibitor were reversed by co-transfection with si-MARCKS. Conclusively, these data confirmed the involvement of miR-34c and MARCKS in the execution of PI3K/AKT pathway in LPS-treated H441 cells.

### 2.8. MiR-34c Could Enhance γ-ENaC Expression and Inhibit the MARCKS Expression in ALI Mouse Model

**Figure 8:**
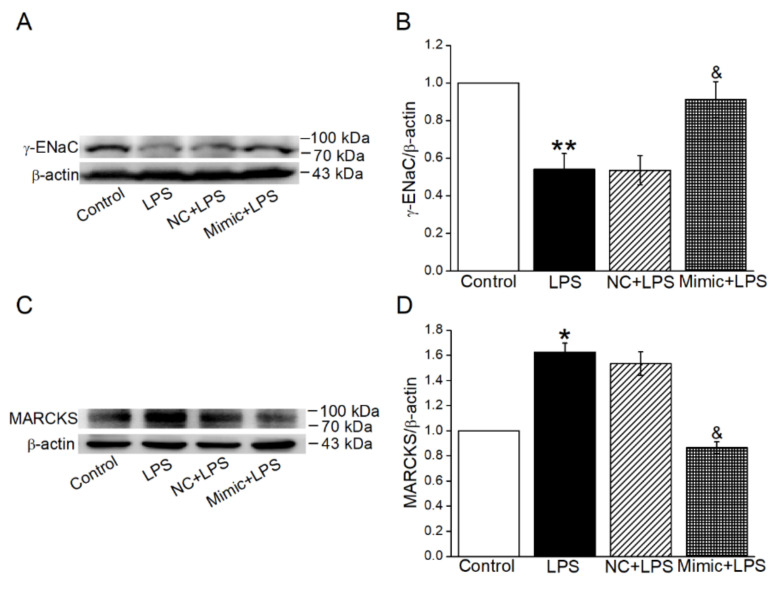
MiR-34c could enhance γ-ENaC expression and inhibit the MARCKS expression in ALI mouse model. (**A**,**C**) Representative blots of γ-ENaC and MARCKS protein in Control, LPS, NC + LPS and Mimic + LPS group in ALI mouse model, respectively. (**B**,**D**) Graphical representation of data obtained from Western blot and quantified through gray analysis (γ-ENaC/β-actin and MARCKS/β-actin) (*n* = 4). Note: * *p* < 0.05, ** *p* < 0.01, compared with Control group; ^&^ *p* < 0.05, compared with NC + LPS group.

To further verify whether miR-34c attenuated lung edema via enhancing γ-ENaC expression, at least partially, through targeting MARCKS in vivo, the Western blot experiment was used to identify the expression of both γ-ENaC and MARCKS in an ALI mouse model. As expected, the protein expression of γ-ENaC was decreased in the LPS group, and this was then reversed by the administration of miR-34c (Figure 8A,B). Meanwhile, miR-34c could inhibit the LPS-enhanced expression of MARCKS, supporting the observation that miR-34c increased γ-ENaC expression by directly targeting MARCKS in ALI mouse model (Figure 8C,D).

## 3. Discussion

Although considerable progress has been made in developing more accurate and effective treatment strategies, the survival rate of ALI remains low [31]. Existing proof has demonstrated that BMSC-sEVs have a protective effect against ALI by inhibiting inflammation, facilitating lung barrier integrity, and eliminating lung edema [32]. The sEVs exert their biological effects by delivering their bioactive substances to recipient cells, among which miRNAs have been extensively studied. In recent years, miRNAs as the therapeutic molecule are impeded by the lack of an appropriate delivery approach, till sEVs emerge as the vehicle to deliver them to recipient cells [33,34]. Moreover, miRNAs are a promising method in regulating the pathways involved in alveolar epithelial ion transport [35]. The removal of lung edema fluid from the alveolar spaces is critically dependent on the ENaC [36]. When the expression of ENaC on the cell membrane increases, the entry of Na^+^ into the alveolar epithelial cells can be promoted by ENaC, and the reabsorption of lung edema begins, followed by the export of Na^+^/K^+^-ATPase on the basement membrane [3]. Previously, we have demonstrated that BMSC-sEVs could upregulate the expression of ENaC, promote lung edema fluid transport and improve the histopathology changes of lung tissue in ALI mice. Recently, reports have highlighted the miR-34c found in BMSC-sEVs [23]. Thus, the objective of the current study is to identify whether BMSC-sEVs can regulate ENaC through transferring miR-34c, thereby exerting protective effect on ALI.

In our experiment, we found that miR-34c performed as an effective miRNA for regulating ENaC expression and a potential treatment strategy in ALI, and this is consistent with the findings of previous studies [37,38]. The human lung adenocarcinoma cell line H441 was one of the classical cell lines for studying the function and activity of ENaC, which had similar biophysical properties with human AT2 cells [39]. We initially identified that the miR-34c could alleviate lung edema and histopathology changes in an LPS-induced ALI mouse model. Then the Western blot and immunofluorescent staining results showed that miR-34c could upregulate ENaC expression in LPS-treated H441 and mouse AT2 cells. Moreover, the qRT-PCR results suggested that miR-34c was highly expressed in BMSCs-CM, which provided a possibility that miR-34c might enhance LPS-inhibited γ-ENaC expression by sEVs. To confirm the above speculation, we labeled the sEVs by PKH26 dye and ascertained that they could be taken up by recipient cells, and as expected, the Western blot assay showed that miR-34c carried by BMSC-sEVs could attenuate LPS-inhibited γ-ENaC expression in alveolar epithelial cells.

By far, the underlying mechanism between miR-34c and γ-ENaC remains unclear. Previous studies and the prediction from the Targetscan website have both implied that miR-34c had many possible targets, such as AREG, MARCKS and so on. Among the potential target genes bound by miR-34c, MARCKS promotes the interaction between PIP2 and ENaC, which positively regulates the channel gating and function accordingly [40,41]. Moreover, MARCKS was proved to be involved in the pathophysiology of various lung diseases [29]. Previous studies have demonstrated that MARCKS inactivation could activate the PI3K/AKT signaling pathway [25,42], which was necessary and sufficient for the elevation of ENaC protein expression [7,26]. The dual luciferase reporter assay and Western blot assay confirmed that miR-34c could specifically bind to MARCKS and that phosphorylated PI3K/AKT protein expression was upregulated. Finally, we verified that miR-34c increased γ-ENaC expression by directly targeting MARCKS in ALI mouse model. Furthermore, it has previously been demonstrated that miR-34c suppressed cell growth in vitro and inhibited tumor growth in vivo by targeting MARCKS, thus suggesting that miR-34c was a novel tumor suppressor in osteosarcoma [43]. In addition, studies have implied that miR-34c directly targeted AREG and downregulated the AREG–EGFR–ERK pathway, which could also regulate ENaC [41].

**Figure 9:**
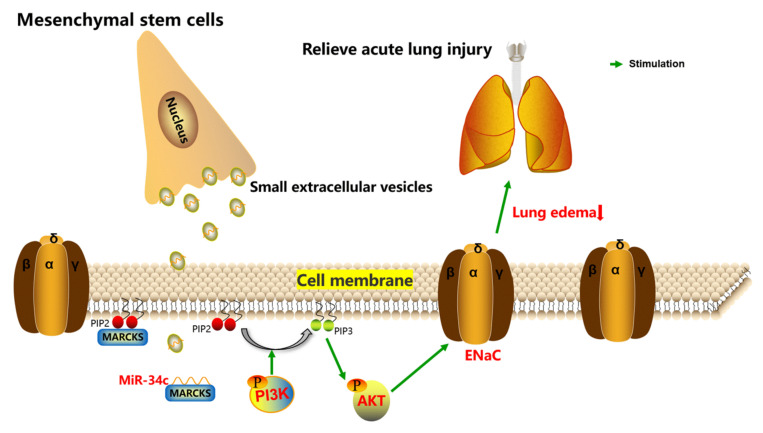
The schematic diagram depicts the potential mechanism about how sEVs derived from MSCs exert their effects on ALI treatment by delivering miR-34c. MSCs can secrete sEVs to transfer miR-34c into alveolar epithelial cells, and miR-34c upregulates ENaC through binding with MARCKS and activating the PI3K/AKT pathway; it also possibly protects against ALI by relieving lung edema. ENaC, epithelial sodium channel; MARCKS, myristoylated alanine-rich C kinase substrate; PI3K, phosphatidylinositol 3-kinase; AKT, protein kinase B; PIP3, phosphatidylinositol 3,4,5-trisphosphate; PIP2, phosphatidylinositol 4,5-bisphosphate.

In the present study, we successfully demonstrated the essential role of MARCKS in regulating expression of γ-ENaC for the first time. Collectively, our study describes a process by which miR-34c derived from BMSC-sEVs can upregulate γ-ENaC via MARCKS and downstream PI3K/AKT pathway (Figure 9). Our data imply that miR-34c in BMSC-sEVs could be a promising strategy in the treatment of ALI.

## 4. Materials and Methods

### 4.1. BMSC Isolation and Culture

BMSCs were isolated from three-week-old pathogen-free male C57 mice, weighing 9–13 g. The mice were anaesthetized by diazepam (17.5 mg/kg, intraperitoneally), followed 6 min later by ketamine (450 mg/kg, intraperitoneally). All experimental procedures were performed according to the guidelines and regulations of Animal Care and Use Ethics Committee and approved by China Medical University (No. CMU2019088). BMSCs were harvested based on the protocol described previously [44]. Then the medullary cavity of femora was washed out by DMEM/F12 medium supplemented with 10% fetal bovine serum (FBS, Gibco, New York, NY, USA), 10 ng/mL recombinant mouse basic fibroblast growth factor (PeproTech, Rocky Hill, NJ, USA), 100 IU penicillin and 100 μg/mL streptomycin. Subsequently, the cells were cultured in a 5% CO_2_ incubator at 37 °C for 24 h, and then the non-adherent cells were removed by changing the culture medium every other day. The identification of BMSCs is shown in Supplementary Figure S1.

### 4.2. Mouse AT2 Cell Isolation and Culture

The lung lobes from newborn C57BL/6J mice within 24 h of birth were carefully separated and minced in PBS. Then the chopped lung tissue was digested with 0.25% trypsin and 0.1% type I collagenase, at 37 °C, for 40 min, in a water bath shaker, respectively. Cells were filtrated and cultured in 5% CO_2_, 37 °C atmosphere with DMEM/F12 medium (containing 10% FBS, 100 IU penicillin and 100 μg/mL streptomycin). The non-adherent cells were collected, and the culture process was repeated 4 times to remove lung fibroblasts. Thereafter, the cell suspension was transferred on the culture dish coated with IgG and incubated for 30 min to remove lymphocytes, macrophages and neutrophils. In the end, the non-adherent cells were placed in the six-well plate, and the medium was changed for the first time after 72 h and then changed every other day. The identification of AT2 cells is shown in Supplementary Figure S2.

### 4.3. BMSC-sEVs’ Collection, Identification and Staining

The BMSCs at passage 2~3 were cultured with serum-free DMEM/F12 medium for 48 h, and then the BMSCs-conditioned medium (BMSCs-CM) was collected and subjected to consecutive centrifugations at 300× *g* (5 min), 2000× *g* (15 min) and 12,000× *g* (35 min) at 4 °C. After that, the supernatant was filtered through a 0.22 μm syringe filter to remove microorganisms and large vesicles in the solution and centrifuged at 150,000× *g* (3 h). The sEVs precipitate were suspended in PBS, and the bicinchoninic acid assay (BCA, Thermo Fisher Scientific, Waltham, MA, USA) was used to detect the concentration of sEVs. The PKH26 dye (Sigma-Aldrich Chemical, Louis, MO, USA) and sEVs were resuspended in Diluent C solution at 37 °C for 5 min, separately. The labeling reaction was stopped by adding 1% BSA (Solarbio, Beijing, China), and sEVs were eluted from the mixture by centrifuging at 100,000× *g* (2 h). Then the labeled sEVs were added to H441 cells and incubated at 37 °C for 20 h. Ultimately, cell nucleus was stained by 4,6-diamidino-2-phenylindole (DAPI).

For transmission electron microscope (TEM) observation, the negative staining was used in the sEVs’ sample preparation. Purified sEVs were fixed with 4% paraformaldehyde for 20 min. After washing with PBS, the sEVs were loaded onto formvar carbon-coated grids and stained with uranyl acetate solution for 5 min. The sEVs sample was dried for 10 min, under room temperature, and then analyzed under a TEM at 80 kV.

### 4.4. CCK-8 Cell Viability Assay

The effects of miR-34c and LPS on cell viability were measured by CCK8 assay. H441 cells were seeded into 96-well plates at a concentration of 1~5 × 104/well and cultured with 10% FBS-containing DMEM/F12 medium in a 5% CO_2_, 37 °C incubator. The cells were randomly divided into 4 groups: cells in the first group were cultured in DMEM/F12 medium (control group, Control), cells in the second group were cultured in LPS containing DMEM/F12 medium (LPS group, LPS) and the cells in the third and fourth group were treated with LPS for 24 h after transfection with miR-34c mimic negative control (Negative Control + LPS group, NC + LPS) and miR-34c mimic (miR-34c mimic + LPS group, Mimic + LPS) for 48 h, respectively. DMEM/F12 medium containing 10% CCK-8 was added to each well. After incubation at 37 °C for 1 h, the optical density value was measured at 450 nm, using a microplate reader, and the cell survival rate was calculated accordingly.

### 4.5. Western Blot Assay

The lung tissues and cells were lysed in RIPA assay buffer (Beyotime, Shanghai, China) and then centrifuged at 12,000× *g* for 10 min. BCA assay kit was used to detect the concentration of protein. Proteins were separated by 10% SDS–polyacrylamide gel electrophoresis and transferred onto the PVDF membrane (Invitrogen, Carlsbad, CA, USA). The blots were blocked with 5% BSA for 1 h, at room temperature, in a shaker, and then incubated with diluted primary antibodies overnight at 4 °C: γ-ENaC (1:2000, Abcam, Cambridge, MA, USA), β-actin (1:2000, Proteintech, Chicago, IL, USA), MARCKS (1:1000, Abcepta, Jiangsu, China), PI3K (1:1000, Affinity, Jiangsu, China), p-PI3K (1:1000, Affinity, Jiangsu, China), AKT (1:1000, Affinity, Jiangsu, China) and p-AKT (1:1000, Affinity, Jiangsu, China). Subsequently, the membrane was washed by TBST three times, 10 min each time. The proteins were incubated with the horseradish peroxidase–conjugated goat anti-rabbit or goat anti-mouse secondary antibody (1:5000, ZSGB-BIO, Beijing, China) at room temperature for 1 h. Finally, the target protein was examined with an ECL kit, using a Tanon-5200 chemiluminescence detection system (Tanon, Shanghai, China), and then quantified by the Image J program.

### 4.6. Quantitative Real-Time PCR

Total RNA was extracted from BMSCs and H441 cells, using the TRIzol reagent (Invitrogen, Carlsbad, CA, USA) according to the manufacture instructions, and the concentration and purity of each RNA sample were quantified by NanoDrop 2000C spectrophotometer (Thermo, Wilmington, DE, USA). MiRNAs were synthesized into cDNA, using a Mir-X miRNA First-Strand Synthesis Kit (TaKaRa, Kusatsu, Shiga, Japan). Quantitative real-time PCR (qRT-PCR) with SYBR Premix Ex Taq II (TaKaRa, Kusatsu, Shiga, Japan) was applied by an Applied Biosystems 7500 Fast Real-Time PCR System (Thermo Fisher, Waltham, MA, USA) with the following primers: miR-34c forward (5′-AGG CAG UGU AGU UAG CUG AUU GC-3′) and reverse (5′-AAU CAG CUA ACU ACA CUG CCU UU-3′). The reaction conditions of the miRNA were a single cycle of 95 °C for 30 s, 5 s at 95 °C and 40 cycles at 60 °C for 30 s. The relative expression of miRNA was analyzed by using the 2^−ΔΔCT^ method, and U6 was used as a reference.

### 4.7. Cell Transfection

H441 and mouse AT2 cells were seeded at 50–60% confluence and cultured in six-well plates. The cells were washed with PBS three times and then incubated with serum-free medium. The MARCKS-siRNA (si-MARCKS), miR-34c mimic (Mimic), miR-34c inhibitor (Inhibitor), miR-34c mimic negative control (NC), MARCKS-siRNA negative control (si-NC), miR-34c inhibitor negative control (Inhibitor NC) and siRNA-mate (GenePharma, Shanghai, China) were transfected into cells according to the manufacturer’s instructions. The final concentration of miR-34c mimic, miR-34c inhibitor and MARCKS-siRNA was 30, 60 and 200 nM, respectively. All transfection reagents were replaced by complete medium after 6 h, and cells were harvested 48 h after transfection for further study.

### 4.8. Immunofluorescent Staining

H441 and mouse AT2 cells were fixed with 4% paraformaldehyde for 30 min at room temperature. Subsequently, the cells were blocked with 5% BSA in PBS for 60 min to block unspecific epitopes and then incubated with γ-ENaC (1:500, Abcam, Cambridge, MA, USA) overnight at 4 °C. Secondary antibodies were diluted in blocking buffer and incubated for 2 h, while being protected from light. Ultimately, the cell nucleus was stained by DAPI for 20 min, and the image was detected by using ×200 magnification.

### 4.9. Dual Luciferase Reporter Gene Assay

The dual luciferase reporter gene assay was used to test whether miR-34c could directly bind to the 3′ untranslated region (3′UTR) of MARCKS, which detected the fluorescence intensity of fluorescein substrate after transfecting cells with a reporter plasmid. H441 cells were seeded at 50–60% confluence in a six-well plate, and then serum-containing medium was replaced by serum-free medium. The 3′UTR of MARCKS (PGLO-MARCKS-wild type (WT)) or the mutant form in which the binding site between MARCKS and miR-34c (PGLO-MARCKS-mutant (MT)) (GenePharma, Shanghai, China) was constructed accordingly. The WT or MT reporter plasmid was transfected into H441 cells with miR-34c mimic (Mimic) or miR-34c mimic negative control (NC) plasmid, respectively. Following transfection for 48 h, the luciferase activity was detected by the Dual Luciferase Reporter Assay Kit (Vazyme, Jiangsu, China) according to the manufacturer’s instructions. Relative luciferase activity was calculated.

### 4.10. ALI Mouse Model Establishment and Lung Wet-to-Dry Ratio Measurement

In order to establish the ALI model, mice were randomly divided into four groups: Control group, ALI model group, ALI model + NC group and ALI model + miR-34c mimic group. ALI model + NC group and ALI model + miR-34c mimic group were anesthetized and injected with negative control (NC) and miR-34c mimic (Mimic) via the caudal vein (2 mg/kg) every 24 h for 3 days, respectively, and then mice were intraperitoneally injected with LPS (5 mg/kg). The Control group was administered an equal volume of normal saline. After a further 24 h, the lung tissues were measured (wet weight) and then placed in a dryer at 80 °C for 48 h to obtain the dry weight. The wet-to-dry (W/D) ratio of each lung tissue was calculated.

### 4.11. Hematoxylin–Eosin Staining

The lung tissues were fixed in 4% buffered paraformaldehyde and cut into sections that were 8–10 µm thick. These sections were then stained with hematoxylin–eosin (HE) for microscope analysis. The lung tissue was scored according to the semi-quantitative scoring system [45], which included four items: alveolar congestion; hemorrhage; infiltration or aggregation of neutrophils in alveolar space or vascular wall, and thickness of the alveolar wall/hyaline membrane formation. The grading scale to evaluate the pathologic findings was as follows: a score of 0 represented none or very minor, 1 represented little or limited, 2 represented intermediate, 3 represented widely distributed or remarkable and 4 represented widespread and severe. The results were scored from 0 to 4 for each item, and four variables were averaged to grade the lung injury score (total score: 0–16).

### 4.12. Statistical Analysis

Data were expressed as the mean ± SE. Normality and homoscedasticity tests were performed by using the Levene and Shapiro–Wilk tests before applying parametric tests. For the comparison of two groups, we used Student’s two-tailed *t*-test; for comparison of multiple groups, we performed ANOVA, followed by Bonferroni’s test, for all groups of the experiment. When the data did not pass the normality or homoscedasticity test, we used a non-parametric *t*-test (Mann–Whitney U-test). Variations were considered significant when the *p*-value was less than 0.05. Statistical analysis was performed with Origin 8.0.

## 5. Conclusions

MiR-34c derived from BMSC-sEVs can attenuate lung edema via enhancing γ-ENaC expression at least partially through targeting MARCKS and activating the PI3K/AKT signaling pathway subsequently.

## Data Availability

The original contributions presented in the study are included in the article/Supplementary Material, and further inquiries can be directed to the corresponding author.

## Supplementary Materials

The following supporting information can be downloaded at: https://www.mdpi.com/article/10.3390/ijms23095196/s1.

## Abbreviations

AKT, protein kinase B; ALI, acute lung injury; AT2 cells, alveolar type 2 epithelial cells; BMSCs, bone marrow mesenchymal stem cells; BMSCs-CM, BMSCs-conditioned medium; BMSC-sEVs, sEVs of bone-marrow-derived MSC; DAPI, 4,6-diamidino-2-phenylindole; ENaC, epithelial sodium channel; LPS, lipopolysaccharide; MARCKS: myristoylated alanine-rich C kinase substrate; miRNA, microRNA; MSC-sEVs, sEVs derived from MSC; PI3K, phosphatidylinositol 3-kinase; PIP2, phosphatidylinositol 4,5-bisphosphate; qRT-PCR, quantitative real-time PCR; sEVs, small extracellular vesicles; TEM, transmission electron microscope.
