# Supplementary material for: Small Extracellular Vesicles Containing miR-34c Derived from Bone Marrow Mesenchymal Stem Cells Regulates Epithelial Sodium Channel via Targeting MARCKS
Source: Int J Mol Sci. 2022 May 6;23(9):5196. doi: 10.3390/ijms23095196 (PMC9101277; doi:10.3390/ijms23095196)

Small extracellular vesicles containing miR-34c derived from bone marrow mesenchymal stem cells regulates epithelial sodium channel *via* targeting MARCKS

Yu Hua, Aixin Han, Tong Yu, Yapeng Hou, Yan Ding, Hongguang Nie*

Department of Stem Cells and Regenerative Medicine, College of Basic Medical Science, China Medical University, Shenyang, 110122, China

*Correspondence: Hongguang Nie, hgnie@cmu.edu.cn

Figure Legends:

Supplementary Figure S1. BMSCs were identified by flow cytometry. **(A, B)** The BMSCs were positive for the cell surface marker CD44 (92.5%), and negative for CD34 (97.3%).

Supplementary Figure S2. AT2 were characterized by immunofluorescence staining. **(A, B)** Nuclei were counterstained by DAPI, and AT2 cell marker surfactant protein C (SP-C) was visualized as green staining. **(C)** The merged image. Scale bar = 50 μm.

Supplementary Figure S1.

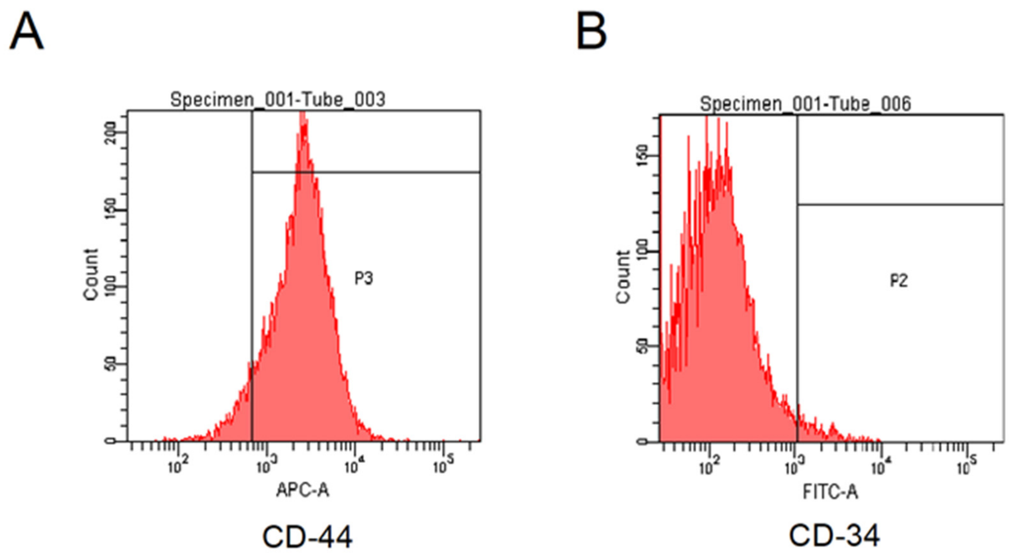

Supplementary Figure S2

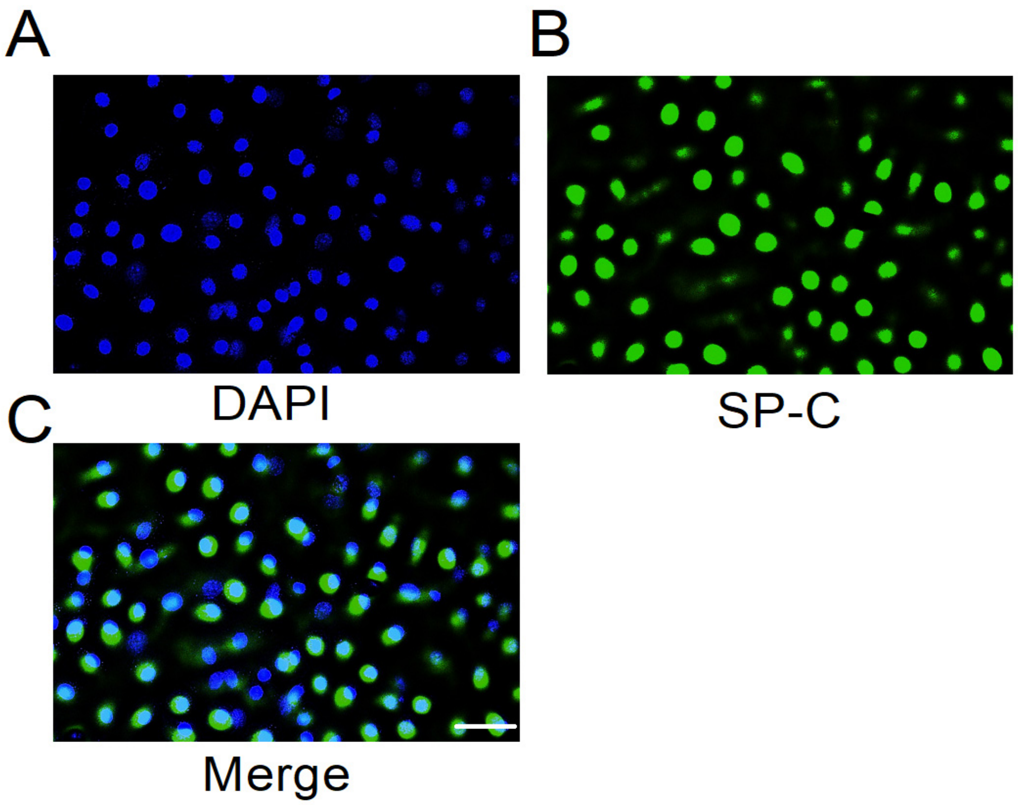

Supplement: Supplementary file 1 [file ijms-23-05196-s001.zip › ijms-1689549-supplementary.pdf]
